# Fatigue among Patients with Type 2 Diabetes Mellitus: The Impact of Spirituality and Illness Perceptions

**DOI:** 10.3390/healthcare11243154

**Published:** 2023-12-12

**Authors:** Maria Vasilaki, Eugenia Vlachou, Anna Kavga, Ourania Govina, Eleni Dokoutsidou, Eleni Evangelou, Anastasia Ntikoudi, Alexandra Mantoudi, Victoria Alikari

**Affiliations:** Post Graduate Program “Management of Chronic Diseases–Diabetes Nursing Care”, Department of Nursing, University of West Attica, 12243 Egaleo, Greece; hn20007@uniwa.gr (M.V.); evlachou@uniwa.gr (E.V.); akavga@uniwa.gr (A.K.); ugovina@uniwa.gr (O.G.); edokout@uniwa.gr (E.D.); elevagel@uniwa.gr (E.E.); antikoudi@uniwa.gr (A.N.); amantoudi@uniwa.gr (A.M.)

**Keywords:** fatigue, illness perceptions, spirituality, type 2 diabetes mellitus

## Abstract

Type 2 Diabetes Mellitus (T2DM) can cause fatigue, negatively affecting the daily functioning and health of individuals. The purpose of this study was to investigate the impact of spirituality and illness perceptions on fatigue among patients with Type 2 Diabetes Mellitus. In this cross-sectional, descriptive study, 100 patients with Type 2 Diabetes Mellitus completed the Fatigue Assessment Scale, the FACIT Sp-12 scale, and the Illness Perception Questionnaire-Revised assessing fatigue, spirituality, and illness perceptions, respectively. The mean age of the sample was 52.18 ± 15.53 years and 65% were insulin-treated patients. The mean score for the FACIT Sp-12 scale was 31.86 ± 7.7, for the FAS 27.0 ± 7.63, and for the Consequences and Emotional Representations of IPQ-R 25.5 ± 5.3. Statistically negative significant correlations were observed between the FACIT Sp-12 total score and the FAS subscales (r = −0.44 to −0.48, *p* < 0.01) and positive correlations between the “IP—Consequences and Emotional Representations” subscales and FAS scores. The total score of the FACIT Sp-12 (β = −0.35) was a negative predictor while Consequences and Emotional Representations (β = 0.28) were positive predictors of the total FAS Score. Participants scored moderate levels of total fatigue. Spirituality and positive illness perceptions may have a protective effect on the fatigue of patients with Type 2 Diabetes Mellitus.

## 1. Introduction

Type 2 Diabetes Mellitus (T2DM) is a chronic disease that affects human health on a global scale [1]. Fatigue, including both mental and physical exhaustion, is a symptom of T2DM which is characterized by a subjective perception of reduced capacity to perform physical, mental, and/or cognitive tasks [2]. The definition of fatigue in patients with T2DM is relatively unclear. Diabetic fatigue syndrome describes the generalized fatigue experienced by patients with T2DM as a multidimensional factor that may persist even after glycemic control is achieved [3]. T2DM and fatigue appear to have a bidirectional relationship fueling and exacerbating each other, thus creating a vicious cycle of diabetic fatigue syndrome. The appearance of fatigue in people with T2DM is related to the shortage of insulin compared to the body’s needs. This deficiency can cause the carbohydrate substrate to be converted into fat. When glycogen stores are depleted, the rate of adenosine diphosphate phosphorylation decreases and thus the rate of adenosine triphosphate synthesis, which acts as a short-term energy store, slows [4]. The correlation between fatigue and factors such as inflammation, body mass index, insulin therapy, and depression is frequently observed in individuals with T2DM and can intensify fatigue symptoms. These conditions frequently co-occur and interact with T2DM, resulting in a multifaceted interaction of organic and psychological factors that contribute to fatigue [5]. In addition, fatigue can be caused by side effects from medications, anxiety about managing the illness, and various short- and long-term complications [6].

As demonstrated in other diseases, the perception of illness is considered a significant determinant of fatigue levels and can lead to adverse health outcomes [7,8]. Individuals who perceive a lack of authority over their health status are prone to encountering higher levels of anxiety and depression compared to those who possess the ability to manage the progression of their disease. The representations of the disease, as well as the perceptions concerning the extent of control, exhibit variations among individuals, even when confronted with identical predicaments [9]. Illness perceptions and their representations include dimensions such as illness identity (the label/name the patient gives to the disease, which is related to the subjectivity of his symptomatology), causes of the disease (data on which the patient bases the etiology of his illness, as well as his/her rate of blame), consequences of the illness, duration of the illness (patients’ perceptions of the duration of symptoms), and control of the illness (perceptions of the effectiveness of treatments) [10]. Broadbent et al. [10] found that patients’ perceptions of T2DM influence their adherence to medication, diet, and body exercise [11]. The positive illness perceptions are positively associated with body pain control, a greater understanding of the disease, and higher levels of perceived quality of life [12]. Illness perceptions, illness representations, and the extent to which they experienced the illness as threatening were predictors of depression in T2DM patients with the dimension of personal control being the strongest dimension [9]. Moreover, there exists a reciprocal association between illness perceptions and fatigue. It is worth noting that fatigue can also influence an individual’s perceptions of their illness. To illustrate, individuals who experience persistent fatigue may cultivate pessimistic attitudes towards their illness, perceiving it as more severe or beyond their control. Consequently, this can exacerbate levels of fatigue and establish a vicious cycle [3].

According to studies [13,14], spirituality, as a source of hope and strength, is a coping strategy for the disease. Healthcare sciences have recognized the spiritual dimension of every human being, which transcends religious bonds and strives for inspiration, respect, meaning, and purpose in life [15]. According to the International Council of Nurses (ICN), nurses have a moral obligation to foster a nurturing atmosphere in which the rights, principles, traditions, and religious beliefs of individuals, families, and communities are upheld [16]. The spiritual dimension of every person seeks answers and comes to the front in cases of illness, intense emotional stress, and difficulties, such as loss, bereavement, and death [17]. The study by Najmeh et al. [18] showed that inner peace and resilience protect T2DM patients from adverse emotions and can result in improved glycemic control. Patients with T2DM who have increased levels of spirituality can optimize T2DM self-management in areas such as regulating blood glucose levels and reducing symptoms [18]. Indian immigrants in Australia believed that their prayers could relieve stress and improve T2DM management. They also held the belief that obtaining blessings from religious leaders could help in the prevention or treatment of illnesses such as T2DM [19]. The presence of high levels of spirituality has been correlated with the development of hope and improved psychological adjustment to illness. Simultaneously, spirituality plays a supportive role in managing self-care and the fear that patients with T2DM may encounter in such circumstances [20]. Generally, spirituality contributes to patient participation in clinical decision-making and higher well-being. Based on the above, healthcare professionals should consider the role that spirituality plays in coping with illness and in increasing patients’ sense of responsibility [21,22].

The purpose of this study was to investigate the impact of spirituality and illness perceptions on the levels of fatigue among patients with T2DM as well as the correlation between the above variables. Studies that simultaneously study fatigue, spirituality, and illness perceptions of T2DM patients are limited, as most studies explore the relationship of fatigue with illness perceptions in different populations [23] or with spirituality [4]. To the best of our knowledge, this study is the first in Greece (or internationally) to study the correlation of fatigue with spirituality and illness perceptions and, also, the first to investigate the above variables among patients with T2DM.

## 2. Materials and Methods

The study design is cross-sectional and descriptive. The sample of the study consisted of 100 patients with T2DM (insulin-treated or not) who were admitted to the hospital setting (convenience sampling). The inclusion criterion was (i) age 18 years old and above. Exclusion criteria were: (i) patients with psychiatric problems, (ii) patients who could not read and understand Greek, (iii) patients with hearing problems, and (iv) patients with severe complications of T2DM such as stage iv diabetic nephropathy or diabetic nephropathy under hemodialysis, diabetic neuropathy, diabetic retinopathy, advanced diabetic vasculopathy as these complications may greatly affect the levels of fatigue. Questionnaires were distributed by the researchers during the patients’ hospitalization. The study was conducted during the period between March 2022 and February 2023. Of the total of 123 hospitalized (during the research period) patients with T2DM, 110 were eligible and 100 accepted to take part in the study (response rate 90.9% among the eligible patients).

To collect the data, the following scales were used:

The Fatigue Assessment Scale (FAS) [24] is a tool for assessing perceived fatigue. It comprises 10 questions on a five-point Likert scale (1 = never–5 = always). The score is derived from the sum of the answers and ranges from 10 to 50 with a higher score indicating a higher level of fatigue. Concerning the domains of the scale, five questions are related to physical fatigue, and five questions to mental fatigue [24,25]. It has been used in many studies in the Greek population among patients on hemodialysis [26,27], patients with osteoarthritis and rheumatoid arthritis [28], and patients with chronic diseases [29] with very good reliability [29]. In this study, Cronbach’s alpha was 0.87, 0.82, and 0.81 for the total FAS, FAS Physical, and FAS Mental, respectively. It can be completed in a few minutes, thus allowing medical and nursing staff to assess fatigue levels and incorporate appropriate interventions to address it in the care provided [29].

The Functional Assessment of Chronic Illness Therapy (FACIT Sp-12) was constructed by Cella et al. [30] and is used to assess spirituality in patients with chronic diseases. It includes three subscales: Meaning in life, Peace, and Faith. Each FACIT Sp-12 subscale contains four questions on a Likert scale of 0 to 4 (0 = no spirituality, 4 = High spirituality). The questions relate to the past seven days. The higher the score, the higher the level of spiritual well-being. The total number of responses provides the overall spiritual well-being information [31]. Its reliability and validity have been tested in several languages [32]. The internal consistency of the Greek version of the scale has been tested (Cronbach’s Alpha Coefficient 0.77) [31]. The internal consistency of the scale in this study was studied: Cronbach’s alpha for total FACIT Sp-12 was 0.81, for Meaning 0.75, for Peace 0.71, and for Faith 0.71).

The Illness Perception Questionnaire-Revised (IPQ-R) measures illness perceptions constructed by Moss-Morris [33] in 2002. The questionnaire consists of nine subscales: (1) Illness Identity-14 items on symptoms, (2) Acute/Chronic Timeline—6 items, (3) Cyclical Timeline—4 items, (4) Consequences—6 items, (5) Personal control—6 items, (6) Treatment Control—5 items, (7) Illness Coherence—5 items, (8) Emotional Representations—6 items, and (9) Causal Representations. The last subscale is structured by 18 items and comprises four additional subscales: (a) Psychological Attributions—6 items, (b) Risk Factors—7 items, (c) Immunity—3 items, (d) Accident οr Chance—2 items. All items are rated on a 5-point Likert-type scale (1 = strongly disagree to 5 = strongly agree). Identity scale items are dichotomized with “Yes” or “No” [33,34]. The internal consistency of the Greek version of the scale has been tested (Cronbach’s alpha coefficients for the subscales 0.6–0.96) [34]. A Cronbach’s alpha value > 0.6 is considered to be within an acceptable range [35]. The current study revealed good reliability with Cronbach’s alpha ranging from 0.64 (domain Causes-External Factors) to 0.89 (domain Illness Coherence).

Also, various sociodemographic and clinical characteristics of the patients were recorded.

To conduct the study, permission was obtained from the Scientific Council (Ethical Committee) of the Hospital (Approval number 11344/16.02.2022). The study was developed following the Declaration of Helsinki, good clinical practice, and all applicable laws and regulations. All participants, before their inclusion in the study, were informed about the purpose of the research, that the anonymity of the data would be ensured, that the data would be used only for the purposes of the research, and that they could withdraw from the study at any time. They were also informed about the law regarding the General Data Protection Regulation (EU) 2016/679, GDPR) and signed a consent document. After completing the questionnaires, the questionnaires were enclosed in a sealed envelope to ensure their safety.

Descriptive statistics were applied to the demographic, clinical, and psychometric characteristics of study participants. For the selection of statistical tests, a normality test was performed, and parametric methods were applied. Relative (%) and absolute frequencies were calculated for qualitative variables, while mean values and standard deviations were calculated for quantitative variables. To test the difference between two independent groups in terms of the mean value (associations of demographic and clinical characteristics on fatigue scores), the *t*-test was applied. Correlations between questionnaires were assessed by calculating Pearson’s r correlation coefficients. Coefficients r < 0.30 were considered negligible, between 0.30–0.50 as weak, between 0.50–0.70 as moderate, between 0.7–0.90 as strong, and >0.90 as very strong [36]. Given the high number of the Greek version of the IPQ-R subscales, best-subset regression analysis was implemented to test which factors predicted fatigue (FAS scores). This was performed with automatic linear regression in which the best subsets were used as a model selection model to reduce the number of predictors. The criteria for entry/removal of each independent variable were based on information criteria (AICC). Data analysis was performed using IBM SPSS^®^, version 28 (IBM Corp. in Armonk, NY, USA).

## 3. Results

Table 1 presents the demographic and clinical characteristics of the sample. Women comprised 59% of the participants, the mean age was 52.18 ± 15.53 years old, while 65% were insulin-treated patients.

Table 2 presents the descriptive characteristics of the scales. The mean total score for the FACIT Sp-12 scale was 31.86 (±7.66), for FAS 27.0 (±7.63), while Physical Fatigue had a higher score (19.66 ± 5.49) than Mental Fatigue (7.34 ± 2.61). The subscales Internal Factors and External Factors had mean 20.30 (±5.45) and 6.33 (±2.21), respectively.

Table 3 shows the correlations with Pearson’s r coefficient between the FACIT Sp-12 and FAS. Negative but weak statistically significant correlations were observed between the FACIT Sp-12 total score and the FAS subscales (r = −0.44 to −0.48, *p* < 0.01). Statistically significant but weak negative correlations were also observed between the FACIT Sp-12 “Meaning” subscale and the FAS subscales (r = −0.28 to −0.31, *p* < 0.05). The negative and statistically significant correlations between Peace and FAS Total Score and Physical Fatigue were negligible (<0.30).

The correlations with Pearson’s r coefficient between the IPQ-R and FAS are presented in Table 4. Statistically significant but weak positive correlations were observed between the “IP—Consequences and Emotional Representations” subscales and FAS scores (r = 0.32–0.46, *p* < 0.01). Also, a negative correlation was observed between the subscale “IP—Illness Coherence” and Mental Fatigue (r = −0.34) (Table 4).

The results of the multiple regression with the FAS total score as a dependent variable are shown in Table 5. For inclusion in the model, a total of 14 variables were considered (age, marital status, insulin-treated or not, T2DM duration, the subscales of the IPQ-R, and the total FACIT Sp-12 Score). The model that was selected based on the Information Criterion (348.84) included six predictors (age 54–87 years old, marital status (married), Total Score of the FACIT Sp-12, IP Consequences and Emotional Representations, Causes—Internal Factors and Behavioral Factors). The model predicted 45.4% of the total variance [F(6, 98) = 6.96, *p* < 0.001]. The most important variable was the total score of the FACIT Sp-12 (0.38) and the least important variable was the marital status (married) (0.07). The total score of the FACIT Sp-12 (β = −0.35) was a negative predictor of the total FAS Score implying that total spirituality may help reduce fatigue levels. IPQ-R subscales Causes—Internal Factors (β = 0.26) and IP—Consequences and Emotional Representations (β = 0.28) were positive predictors of total FAS Score meaning that these subscales may contribute to increased levels of fatigue.

As for the results of the multiple regression with Physical Fatigue as the dependent variable for inclusion in the model, a total of 14 variables were considered (age, marital status, insulin intake, the subscales of the IPQ-R, and the total FACIT Sp-12 score). The model that was selected based on the Information Criterion (288.75) included five predictors (age 54–87 years old, Total Score of the FACIT Sp-12, IP Consequences and Emotional Representations, Causes—Internal Factors and Behavioral Factors). The model predicted 41.5% of the total variance [F(5, 98) = 14.92, *p* < 0.001]. The most important variable was the total score of the FACIT Sp-12 (0.51) and the least important variable was age (0.08). The total score of the FACIT Sp-12 (β = −0.37) was a negative predictor of the Physical FAS Score implying that total spirituality may help reduce physical fatigue levels. The IPQ-R subscales Causes—Internal Factors (β = 0.24) and IP—Consequences and Emotional Representations (β = 0.26) were positive predictors meaning that these subscales may contribute to increased levels of physical fatigue.

Regarding the multiple regression with Mental Fatigue as the dependent variable, a total of 14 variables were considered (age, marital status, insulin intake, the subscales of the IPQ-R, and the Total FACIT Sp-12 Score). The model that was selected based on the Information Criterion (288.75) included five predictors (years from T2DM diagnosis, Total Score of the FACIT Sp-12, Married, IP Timeline Cyclical, and Causes—Internal Factors). The model predicted 40.1% of the total variance [F(5, 98) = 14.11, *p* < 0.001]. The most important variable was the total score of the FACIT Sp-12 (0.47) and the least important variable was the marital status (0.05). The total score of the FACIT-Sp-12 (β = −0.40) was a negative predictor of the mental FAS score meaning that total spirituality may help reduce mental fatigue levels. The IPQ-R subscales Causes—Internal Factors (β = 0.31) and IP Timeline Cyclical (β = 0.20) were positive predictors meaning that these subscales may contribute to increased levels of mental fatigue (Table 5).

Regarding scores on the FACIT Sp-12, IPQ-R, and FAS subscales between insulin-treated and non-insulin-treated patients, insulin-treated participants scored higher (Mean: 26.29, SD:5.46) on the IPQ-R “IP Duration of Illness, Acute/Chronic” subscale than non-insulin-treated patients (Mean: 23.91, SD:5.49), (t(98) = 2.07, *p* = 0.04). Patients with hypertension had a higher total FAS score (Mean:29.00, SD:7.64) and a higher score FAS Physical subscale (Mean: 21.42, SD: 5.52) than those without hypertension (*p*< 0.05). Τhere was no statistically significant difference in total fatigue levels between insulin-treated (Mean: 27.20, SD ± 7.85) and non-insulin-treated patients (Mean: 26.89, SD ± 7.56, (t(98) = 0.19, *p* = 0.85) (Table 6).

## 4. Discussion

This study was conducted in the Attica Region, the most populated area of Greece. The aim was to determine the predisposing factors of fatigue among 100 patients with Τ2DM. Although several studies [37,38] have explored the broader impact of spirituality on overall well-being and quality of life in individuals with chronic illnesses, research specifically focusing on the relationship between spirituality, illness perceptions, and fatigue, is limited. As far as we know, it is the first study not only in Greece but also internationally that correlates the variables of fatigue, spirituality, and illness perceptions in patients with T2DM. This study is significant because the investigation of the role of spirituality and illness perceptions in fatigue, as well as the interaction of the above variables, enables the study of the predisposing factors that lead to fatigue and poor health outcomes.

According to our findings, participants experienced moderate total fatigue, while levels of physical fatigue were higher than levels of mental fatigue. Among individuals with T2DM, fatigue is a prevalent symptom that poses a significant challenge to the adoption of healthy behaviors. While fatigue can also manifest in other chronic conditions, its impact appears to be more pronounced in individuals living with diabetes [39]. A phenomenological study conducted among individuals diagnosed with T2DM, who have experienced diabetes-related fatigue, has highlighted the detrimental consequences it has on various aspects of their lives, particularly their overall well-being, ability to perform daily tasks, and financial stability [40].

The mean total score of the spirituality scale was found to be satisfactory. Also, a satisfactory score was found for the dimensions of the spirituality scale: Peace, Meaning, and Faith. Regarding illness perceptions, participants scored moderately on both the total scale score and individual dimensions.

The present study found that spirituality is positively related to physical and mental fatigue, which means that spirituality acts protectively on the fatigue of patients with T2DM. The positive correlation between spirituality and fatigue in patients with Τ2DM found in the present study is consistent with the literature [4]. A study [4] conducted in Indonesia on patients with T2DM showed that a program implemented through the Islamic religion (a combination of stress management, prayer, and mindfulness) resulted in empowering patients and therefore, increased their resilience and reduction of physical and mental fatigue. The research conducted by Lucchetti et al. [41] underscores the significance of spirituality in individuals diagnosed with T2DM. Their findings indicate that individuals with higher levels of spirituality tend to exhibit lower levels of depression and anxiety symptoms. Additionally, the study reveals that spirituality acts protectively against the adverse psychological effects of stressors related to T2DM. Notably, a noteworthy correlation is observed between spirituality and the comprehensive quality of life, encompassing physical, psychological, and social well-being. This suggests that spirituality plays a pivotal role in enhancing the overall well-being of individuals living with T2DM [42]. The study conducted by Onyishi et al. [20] revealed that higher levels of spiritual well-being were associated with lower levels of fatigue. The researchers posited that spirituality could potentially function as a coping mechanism, endowing individuals with a profound sense of purpose and significance, thereby mitigating the manifestation of fatigue symptoms. Another study [43] explored the role of spirituality in the management of fatigue among patients with different chronic illnesses, including diabetes. The results of this study revealed a significant association between participation in spiritual activities, such as prayer and meditation, and reduced levels of fatigue. The researchers put forth the hypothesis that spirituality might enhance psychological well-being and resilience, thereby leading to improved fatigue management. In a qualitative study [44], although diabetes was not specifically examined, the results indicated that spirituality was associated with decreased levels of fatigue across various chronic conditions. The authors of this review emphasized the significance of addressing spiritual needs within healthcare settings in order to enhance overall well-being and symptom management [44]. At this point we should emphasize that spirituality should not be seen as a substitute for medical treatment or interdisciplinary healthcare. It should be considered as a complementary approach that can support individuals in their overall well-being and coping strategies.

Illness perceptions refer to an individual’s beliefs, thoughts, and understanding of their illness, including their perceptions of its causes, consequences, and controllability. These perceptions can significantly influence how patients experience and cope with fatigue. Our study showed that patients’ illness perceptions of T2DM are related to fatigue levels. Similarly, a study [45] conducted among patients with T2DM revealed that patients who had a positive perception of their disease had significantly lower levels of fatigue [45]. It has been suggested that the perception of illness plays a role in the coping and management of illness, which in turn influences health outcomes. Another study [46] conducted in Greece revealed that the individuals with Τ2DΜ who reported that they had their disease under control experienced significantly reduced levels of fatigue compared to those who did not [46]. This suggests that negative illness perceptions can contribute to increased fatigue in patients with diabetes and highlights the importance of addressing and modifying negative illness perceptions to alleviate fatigue symptoms. On the other hand, patients who have more positive illness perceptions may be more motivated to engage in self-care behaviors, such as regular exercise, healthy eating, and medication adherence, which can help reduce fatigue symptoms. Modifying illness perceptions through interventions, such as cognitive-behavioral therapy or psychoeducation, may have a positive impact on reducing fatigue in patients with T2DM. In general, although there is supporting evidence indicating a connection between how patients with T2DM perceive their illness and the experience of fatigue, the precise dynamics and intensity of this correlation may differ based on various factors, including individual attributes.

Regarding the effect of age in the present study, it was found age was a negative predictor of both total and physical fatigue. In the literature, it has been found that fatigue in patients with T2DM increases with age [47]. Older patients significantly limit their activities because they get tired more easily than younger patients, causing distress, frustration, and eventually depression [48]. Spirituality is a component that assists them in facing a new situation and overcoming their fears. They also increase their attendance at religious places and become more intimate with the divine [49].

In our study, although marital status was the least significant variable that could affect fatigue levels, we should emphasize that it is one of the predisposing factors for fatigue. Support provided by family members to patients with T2DM is very important due to the various limitations of the disease. Patients who live in a family setting demonstrate significantly reduced levels of fatigue, primarily due to the assistance they receive in their daily lives [50]. In contrast, patients who live alone are unable to share the burden of the illness or share their worries with other members of the family. Thus, attempting to manage the illness on their own, leads to increased levels of fatigue [51].

As for the limitations of the study, the questionnaires were completed during the patients’ hospitalization; thus, the responses may have been influenced by the comorbidity, the presence of other patients, family members, and medical-care professionals. Also, the study sample came from a single hospital. Therefore, it is difficult to generalize the results. The convenience sampling method was chosen due to the easy access to information as the researchers could collect a considerable amount of data within a short period of time.

Also, patients with severe comorbidities (diabetic nephropathy under hemodialysis, diabetic neuropathy, diabetic retinopathy, advanced diabetic vasculopathy) were excluded from the study since these variables may importantly affect the levels of fatigue. This research question could be the subject of another future study.

## 5. Conclusions

Patients with T2DM experienced satisfactory levels of spirituality, moderate levels of illness perceptions, and moderate levels of total perceived fatigue. They were also, found to have higher physical and lower mental fatigue. Positive illness perceptions, high Spirituality, and age > 54 were found to have a protective effect on fatigue in patients with T2DM. Based on the above information, it is considered that early detection and evaluation of fatigue is essential for patients with T2DM. Due to the complexity of the pathogenesis of fatigue, it is challenging for healthcare professionals to effectively intervene. It is essential for healthcare professionals working in the clinical setting to recognize fatigue-related issues in order to assist patients in coping with measures. It is rather significant the recognition of patients who may be at risk of developing fatigue (those who live alone, and those with low spirituality and negative illness perceptions). The educational interventions of health professionals that focus on the adoption of positive illness perceptions can help patients develop appropriate T2DM self-management strategies. Integrating spirituality into healthcare discussions can enhance patient care and well-being, although further research is necessary to fully understand the extent of its impact. Healthcare practitioners should offer comprehensive education during counseling sessions to assist patients in comprehending the correlation between fatigue and diabetes. This knowledge will enable patients to make well-informed decisions regarding lifestyle modifications, medication adherence, and coping strategies. The implementation of Cognitive-Behavioral Therapy has the potential to be advantageous in addressing illness perceptions and fatigue among individuals with T2DM. It is crucial for healthcare providers to motivate patients to engage in personalized exercise programs that consider their individual capabilities and preferences. To effectively address both the physical and psychological aspects of fatigue and illness, an integrated care approach should be adopted, involving collaboration among healthcare professionals from various disciplines such as endocrinologists, psychologists, and dietitians. Policies should prioritize patient-centered care, which can be achieved through shared decision-making, patient education, and self-management support. Exploring the relationship between fatigue and spirituality in further studies could reveal how spiritual beliefs might mitigate the effects on patients’ well-being. Moreover, delving into how people perceive the causes and outcomes of T2DM may yield valuable knowledge about the psychological determinants of coping mechanisms for this condition.

## Figures and Tables

**Table 1:** Demographic and clinical characteristics of the sample (*n* = 100).

	*n*	%
Gender		
Male	41	41.0
Female	59	59.0
Place of Residence		
Rural	8	8,0
Semi-urban	28	28.0
Urban	64	64.0
Marital Status		
Single	27	27.0
Married	51	51.0
Divorced	13	13.0
Widowed	9	9.0
Educational Level		
Basic-primary School	8	8.0
Secondary School	13	13.0
High School	42	42.0
University Student	9	9.0
University Graduate	28	28.0
Occupational Status		
Unemployed	8	8.0
Housemaking	9	9.0
Self-employed	6	6.0
Private Employee	21	21.0
State Employee	33	33.0
Retired	20	20.0
Student	2	2.0
Hypertension		
No	52	52.0
Yes	48	48.0
Rheumatological Diseases		
No	82	82.0
Yes	18	18.0
Insulin		
No	35	35
Yes	65	65
Age (years)	Mean (±SD)	
	52.18 ± 15.53	
Duration of T2DM (Years)	8.87 ± 9.08	

SD: Standard Deviation.

**Table 2:** Descriptive characteristics of the scales.

	Min	Μax	Mean	SD
Total FACIT Sp-12	10.00	44.00	31.86	7.66
Meaning	1.00	15.00	10.62	2.18
Peace	4.00	15.00	9.21	2.70
Faith	3.00	16.00	10.65	3.59
Total FAS Score	10.00	41.00	27.00	7.63
Physical	7.00	30.00	19.66	5.49
Mental	3.00	14.00	7.34	2.61
IPQ-R				
Causes—Internal Factors	9.00	33.00	20.30	5.45
Causes—Behavioral Factors	3.00	14.00	8.39	2.68
Causes—External Factors	3.00	11.00	6.33	2.21
IP—Consequences and Emotional Representations	16.00	35.00	25.47	5.28
IP—Timeline Acute/Chronic	11.00	35.00	24.74	5.57
IP—Treatment Control	5.00	20.00	14.61	2.63
IP—Personal Control	11.00	29.00	20.59	3.41
IP—Illness Coherence	4.00	20.00	15.48	3.32
IP—Timeline Cyclical	4.00	11.00	8.04	1.50

SD: Standard Deviation; IP = Illness Perceptions.

**Table 3:** Correlations between fatigue scale and spirituality scale.

	FAS Total	FAS Physical	FAS Mental
FACIT Sp-12 Total	−0.48 **	−0.44 **	−0.48 **
FACIT Sp-12 Meaning	−0.31 **	−0.30 *	−0.28 *
FACIT Sp-12 Peace	−0.25 *	−0.25 *	−0.19 *
FACIT Sp-12 Faith	−0.13 *	−0.11 *	−0.14 *

** *p* < 0.01. * *p* < 0.05.

**Table 4:** Correlations between fatigue scale and illness perceptions scale.

	Causes—Internal Factors	Causes—Behavioral Factors	Causes—External Factors	FAS Total	FAS Physical	FAS Mental
IP—Consequences and Emotional Representations	0.52 **	−0.04	−0.01	0.44 **	0.46 **	0.32 **
IP—Timeline Acute Chronic	0.10	0.11	−0.17	0.11	0.09	0.15
IP—Treatment Control	−0.21 *	0.09	−0.16	−0.26 **	−0.23 *	−0.29 **
IP—Personal Control	0.03	0.24 *	−0.03	−0.19	−0.19	−0.17
IP—Illness Coherence	−0.16	−0.04	−0.12	−0.25 *	−0.19	−0.34 **
IP—Timeline Cyclical	0.22 *	−0.14	0.06	0.20 *	0.17	0.25 *
Causes—Internal Factors	-	0.23 *	0.17	0.47 **	0.46 **	0.40 **
Causes—Behavioral Factors		-	0.35 **	0.18	0.20 *	0.12
Causes—External Factors			-	0.09	0.12	0.02
FAS Total Score				-	0.97 **	0.88 **
FAS Physical					-	0.74 **
FAS Mental						-

** *p* < 0.01. * *p* < 0.05, IP = Illness Perceptions.

**Table 5:** Multiple regression with the FAS Score as dependent variable.

	B	SE B	Β	Importance
Total FAS score				
(Constant)	19.00	5.35		
FACIT Sp-12 Total	−0.34	0.09	−0.35 ***	0.38
Causes—Internal Factors	0.36	0.13	0.26 **	0.20
IP—Consequences and Emotional Representations	0.39	0.15	0.28 **	0.18
Causes—Behavioral Factors	0.43	0.23	0.15	0.09
Age between 54 and 87 years old Younger ^a^	−2.06	1.18	−0.14	0.08
Married Not married ^a^	−2.35	1.39	−0.16	0.07
Physical Fatigue				
(Constant)	14.53	3.51		
FACIT Sp-12 Total	−0.26	0.06	−0.37 ***	0.51
IP-Consequences and Emotional Representations	0.26	0.10	0.26 *	0.17
Causes—Internal Factors	0.24	0.10	0.24 *	0.15
Causes—Behavioral Factors	0.31	0.16	0.15	0.09
Age between 54 and 87 years old Younger ^a^	−1.61	0.86	−0.15	0.08
Mental Fatigue				
(Constant)	6.57	1.53		
FACIT Sp-12 Total	−0.14	0.03	−0.40 ***	0.47
Causes—Internal Factors	0.15	0.04	0.31 ***	0.29
IP-Timeline Cyclical	0.34	0.14	0.20 *	0.12
Diabetes diagnosis 6–40 years	−0.76	0.41	−0.15	0.07
Married	−0.69	0.44	−0.13	0.05

Dependent Variables: Total FAS Score; R^2^ = 0.45 (*p* < 0.001), Physical Fatigue; R^2^ = 0.41 (*p* < 0.001), Mental Fatigue, R^2^ = 0.40 (*p* < 0.001); * *p* < 0.05, ** *p* < 0.01, *** *p* < 0.001; IP = Illness Perceptions, ^a^ = reference group.

**Table 6:** Mean scores of the FAS based on the presence of insulin treatment and hypertension.

Insulin-Treated		Mean	SD	t(98)	*p*
FAS Physical	Yes	19.83	5.52	0.22	0.82
	No	19.57	5.51		
FAS Mental	Yes	7.37	2.85	0.09	0.93
	No	7.32	2.49		
FAS Total	Yes	27.20	7.85	0.19	0.85
	No	26.89	7.56		
Hypertension					
FAS Physical	Yes	21.42	5.52	3.22	0.00
	No	18.04	4.99		
FAS Mental	Yes	7.58	2.56	0.90	0.37
	No	7.12	2.65		
FAS Total	Yes	29.00	7.64	2.59	0.01
	No	25.15	7.20		

SD: Standard Deviation.

## Data Availability

Data are available from the corresponding author upon reasonable request.
